# Harman and norharman, metabolites of entomopathogenic fungus *Conidiobolus coronatus* (Entomopthorales), disorganize development of *Galleria mellonella* (Lepidoptera) and affect serotonin-regulating enzymes

**DOI:** 10.1371/journal.pone.0204828

**Published:** 2018-10-03

**Authors:** Anna Katarzyna Wrońska, Mieczysława Irena Boguś, Agata Kaczmarek, Michalina Kazek

**Affiliations:** 1 Witold Stefański Institute of Parasitology, Polish Academy of Sciences, Warsaw, Poland; 2 BIOMIBO, Warsaw, Poland; Natural Resources Canada, CANADA

## Abstract

Naturally occurring entomopathogenic fungi such as *Conidiobolus coronatus* are important regulatory factors of insect populations. GC-MS analysis of fungal cell-free filtrates showed that *C*. *coronatus* synthesizes two β- carboline alkaloids: harman and norharman. Significantly higher levels of both alkaloids are produced by *C*. *coronatus* in minimal postincubation medium than in rich medium. The beta-carboline alkaloids may have an effect on the nervous system of insects and their behavior. Harman and norharman were applied to *Galleria mellonella* larvae (a parasite of honeybees) either topically or mixed with food. Larvae received alkaloids in three concentrations: 750, 1000 or 1250 ppm. The effect on the survival and further development of larvae was examined. Both harman and norharman delayed pupation and adult eclosion, and inhibit total monoamine oxidase activity. In addition, they increased the serotonin concentration and decreased the monoamine oxidase A level in the heads of the moths. It is likely that the alkaloids were metabolized by the insects, as their effect wore off 24 hours after topical application. This is the first study to show that *C*. *coronatus* produces alkaloids. Its aim was to identify the actions of β-carboline alkaloids on insect development and serotonin-regulating enzymes. Knowledge of the potential role of harman and norharman in the process of fungal infection might lead to the development of more effective and environmentally-friendly means of controlling insect pests.

## Introduction

Classical pesticides are frequently used to reduce the abundance of harmful insect populations; however, their excessive and widespread application results in a loss of biodiversity, and hence interest has grown in identifying natural enemies of insects for use as control agents [1]. Entomopathogenic fungi have the potential to play a vital role as biological control agents of insect populations [2,3]. Among the many species of fungi capable of infecting insects, the cosmopolitan soil fungus *Conidiobolus coronatus* (Zygomycota, Entomophthorales) is known to be an opportunistic entomopathogen causing high and rapid insect mortality, and acting specifically with a narrow host range [4, 5]. However, although the *C*. *coronatus* genome [6] has been sequenced, little is known of the toxic substances by which the fungus kills its victims. Previous studies have shown that *C*. *coronatus* produces several insecticidal proteins which can incapacitate the immunocompetent cells known as hemocytes [3, 7, 8]. However, no information is available about the effects of the low-molecular weight metabolites of *C*. *coronatus* (for example, alkaloids) which may have particularly interesting effects on the nervous system.

Regarding their biological effects, β-carboline alkaloids may interact selectively with specific enzymatic targets, resulting in a variety of pharmacological effects. *In vivo* and *in vitro* studies have offered some insight into the potency, selectivity and modes of action of harman and norharman with regard to inhibiting monoamine oxidase A (MAO-A) and monoamine oxidase B (MAO-B) in vertebrates. The inhibition of the two MAO isoenzymes by the harmala alkaloids was found to be influenced by the degree of saturation of its six-membered ring containing one nitrogen atom and substituents of the β-carboline system [9)] MAO catalyzes the oxidative deamination of 5-hydroxytryptamine (5-HT; serotonin) by converting it into 5-hydroxy-3-indolacetaldehyde (5-HIAL), which is further processed into 5-hydroxy-3-indolacetic acid (5-HIAA) by aldehyde dehydrogenase. Although MAO-A exhibits the highest preference for 5-HT and catalyzes its metabolism under physiological conditions, MAO-B is the only MAO isoenzyme that has been identified in 5-HTergic neurons [10]. MAO enzymes are known to be present in the insect brain [11].

Serotonin has been examined qualitatively and quantitatively in the central nervous system (CNS) of many insect species. In insects, the synthesis of 5-HT from tryptophan via 5-hydroxytryptophan is catalyzed by tryptophan hydroxylase followed by DOPA/5-hydroxytryptophan decarboxylase [12]. Serotonin plays a key role in regulating and modulating physiological and behavioral processes in insects. In *Drosophila melanogaster*, elevated 5-HT levels were found to significantly increase periods of sleep [13], but also inhibited entrainment of the *Drosophila* circadian clock to light [14]. 5-HT has been demonstrated to modulate appetite in several insect species. For example, 5-HT injection in the hemolymph decreased feeding in another dipteran species, the flesh fly *Neobellieria bullata* [15]. In the honey bee, injection into the brain inhibited feeding and injection into the gut excited muscle contractions, although general elevation of 5-HT in bee hemolymph did not affect food intake [16]. In the yellow-fever mosquito *Aedes aegypti*, blood-feeding success was reduced by administration of the 5-HT-depleting drug α-methyl-tryptophan [17]. Finally, 5-HT has also been found to modulate several insect behaviors through influencing some aspects of learning and memory. Flies with genetically or pharmacologically-reduced 5-HT levels in the brain demonstrated strongly reduced memory formation in a behavioral test where they were trained to avoid a chamber position associated with high temperature [18].

As well as in mammals, harman and norharman can also act as MAO inhibitors in invertebrates. Therefore, these alkaloids have the ability to dysregulate various insect physiological processes by influencing the level of serotonin.

The present work demonstrates that the entomopathogenic fungus *C*. *coronatus* produces two β-carboline alkaloids, harman and norharman, which influence the development of the wax moth *Galleria mellonella* (Lepidoptera, Pyralidae) by affecting serotonin-regulating enzymes. The moth is commonly used as a model organism for *in vitro* toxicology and pathogenicity testing [19].

## Materials and methods

### Fungus

The *Conidiobulus coronatus* (isolate number 3491) used in the study, originally isolated from *Dendrolaelaps* spp., was obtained from the collection of Prof. Bałazy (Polish Academy of Sciences, Research Center for Agricultural and Forest Environment, Poznań). *C*. *coronatus* was routinely cultured on Sabouraud agar medium (SAM) with the addition of homogenized *G*. *mellonella* larvae at a final concentration of 10% wet weight (SAM-G) to enhance the sporulation and virulence of the fungus. SAM was prepared by dissolving powdery Sabouraud 4% Dextrose Agar (Merck Millipore) in ultra-pure water. Frozen *G*. *mellonella* larvae were homogenized and added to the liquid medium. The whole medium was sterilized. SAM-G was poured into sterile Petri dishes. The fungal cultures were maintained at 20°C with periodic changes of light (12 hours of light: 12 hours of dark).

Seven-day-old cultures were rinsed with sterile water to harvest the conidia, and 100 μl portions of the suspension, each containing an average of 52 conidia, were used for the inoculations. The number of conidia on the Petri dishes were calculated using microscopy. Conidia were recovered from the sporulated fungus and suspended in sterile water, following which, they were counted in a Thoma chamber. In order to obtain the mixture of alkaloids, *C*. *coronatus* was cultivated at 20°C in 500 ml Erlenmeyer flasks containing 250 ml of minimal medium (MM) or rich medium (LB) as described by Bania et al., [20] but without shaking. Three weeks after inoculation, the mycelia were removed by filtration through Whatman no. 1 filter paper. The cell-free filtrates were used for GC-MS analysis.

### Determination of harman and norharman

The filtrates were extracted by solid phase extraction using SmartPrep (Horizon). A volume of 250 ml of filtrates was used for the extraction in 3 independent replicates. Waters OASIS MCX 8 150 mg cartridges were conditioned with 5% ammonia water in methanol and methanol. The loaded samples were eluted with 10 ml methanol (1ml/min), and the extract was evaporated under nitrogen to dry weight. The efficiency of this extraction method was 85%. A concentration of 1 mg/ml was prepared for GC-MS (gas chromatography mass spectrometry) analysis.

The GC-MS analyses were carried out on a GC-2010 Plus chromatograph coupled with a mass detector (GCMS-QP2010, Shimadzu). Helium was used as the carrier gas. A ZB-5 MS (Zebron, Phenomenex) column (lenght 60.0m, thickness 0.25μm, diameter 0.25mm) was used. The column oven temperature cycle was 80°C for three minutes, then 80°C to 310°C at 4°C/minute; the final temperature was held for 10 minutes. The column flow was 1.0ml/min. The split was 10:1. The ion source temperature was 200°C and the interface temperature 310°C. Samples were run in the scan mode. The quantities of harman and norharman were determined by an internal standard. Harman (Sigma-Aldrich, purity 98%, catalog number: 103276) and norharman (Sigma-Aldrich, purity 98%, catalog number: N6252) were used as standards. Both substances were applied at a concentration of 3 ng/ml of dichloromethane (for gas chromatography MS SupraSolv, Sigma-Aldrich). The analyses were carried out in three independent replicates.

### Insects

*G*. *mellonella* (Lepidoptera) was reared on an artificial diet prepared according to Sehnal’s recipe [21] in constant darkness at a temperature of 30°C and humidity of 70% r.h. Fully-grown larvae were collected before pupation, surface-sterilized, homogenized and used as a supplement in the fungal cultures. The larvae were also used in the virulence tests which were routinely performed after each fungus transfer [22]. Three-day-old final instar (7^th^ instar) larvae were used to determine the influence of β-carboline alkaloids on insect development, concentration of serotonin and MAO-A in heads, as well as MAO activity in head capsules.

### Harman and norharman application

Harman and norharman (Sigma-Aldrich) were administered to the *G*. *mellonella* larvae by topical application and by the diet at final concentrations of 750 ppm (1H, 1N), 1000 ppm (2H, 2N) and 1250 ppm (3H, 3N) (H-harman, N-norharman) according to Bouayad et al. [23]. The alkaloids were diluted in acetone for topical applications, where each larva received 5μl of harman or norharman solution containing 750, 1000 or 1250 ppm of the appropriate alkaloid. Therefore each larva received 3.75μg, 5 μg or 6.255 μg topically. For intake with food, harman and norharman were dissolved in 5% methanol in distilled water, and 5 ml of this alkaloid solution was incorporated into 5 g of insect food to achieve a final concentration of 750, 1000 or 1250 ppm. Therefore 1g of insect food contained 750 μg, 1000 μg or 1250 μg. The solvent was then evaporated from the food at 35°C in an oven for 48 hours. Addition of alkaloids to food did not significantly affect the pH value. The pH of the control food was 5.83±0.04. While in food with the addition of alkaloids, this parameter was: 1H- 5.79±0.07; 2H- 5.82±0.07; 3H- 5.80±0.04; 1N- 5.81±0.02; 2N- 5.81±0.06; 3N- 5.81±0.02. The larvae consumed the treated food throughout the entire experiment. The control group consisted of insects fed with food treated with solvent instead of alkaloids. For harman and norharman application (topical and with food) three-day-old final instar (7^th^) larvae were selected. Insects in this developmental stage were so large that it was possible to precisely collect the material for testing. In addition, the larvae were still feeding: feeding ceases on the 5^th^-6^th^ day of the final instar [24].

### Examination of the effect on larval development

The development of treated larvae (10 individuals per Petri dish) was monitored each day, together with larval mortality, number of pupae and emerged adults. Ten replicates were performed for treated and control larvae. Larvae were fed appropriate food and maintained at standard temperature and humidity (30°C and humidity of 70% r.h) throughout the experiment.

### Measurements of serotonin (5-HT) and monoamine oxidase (MAO) level in larval heads

Serotonin and MAO-A concentration and MAO-A activity in heads of *G*. *mellonella* larvae was checked 1 and 24 hour after topical application of alkaloids and 24 hours after ingestion of food mixed with alkaloids. The treated and control larvae were placed on ice for five minutes and quickly decapitated. The heads were then homogenized in 100 μl of insect physiological saline (IPS) with phenylthiourea (PTU) in order to inhibit melanization. Twenty larvae were used for the preparation of each sample.

Total MAO activities were measured using MAO-Glo (Promega Corporation, USA) kit. The assays were performed by incubating the MAO enzymes with a luminogenic MAO substrate, a derivative of beetle luciferin. The amount of MAO-A was determined using MAO A ELISA Kit (SunRed Biotechnology Company, Shanghai, China) and the amount of 5-HT was examined using Serotonin ELISA Fast Track (Labor Diagnostika Nord, Germany). Each test was performed in three independent replicates.

### Statistics

STATISTICA 6.1 software (StatSoftPolska) was to evaluate the of normality of the data and its statistical significance. The Kolmogorov-Smirnov test was used to check normality. The t-test for independent samples was used to compare the results of the control group and the study group. The results were regarded as being statistically significant at P ≤ 0.05.

## Results

### Harman and norharman produced by C. *coronatus*

**Fig 1:**
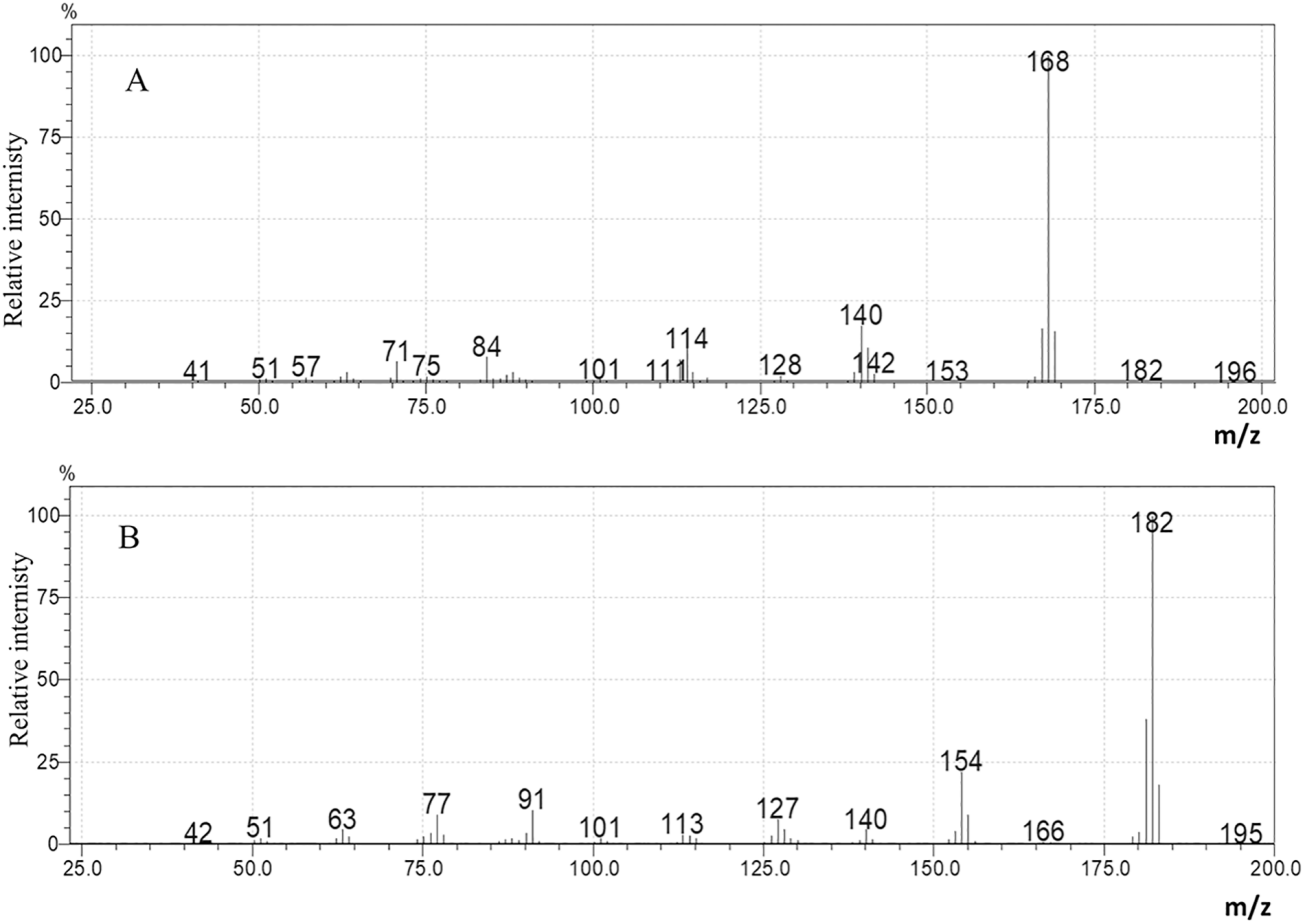
Mass spectra of norharman (A) and harman (B).

*C*. *coronatus* was cultured in minimal medium (MM) and rich medium (LB) for three weeks. The cell-free fungal filtrates were analyzed by GC-MS as described in materials and methods. Sterile MM and LB media were used as controls.: Norharman (M + *m/z* = 168; retention time 20.75min) and harman (M + *m/z* = 182; retention time 20.65min) were identified by mass spectrometry (Fig 1).

**Fig 2:**
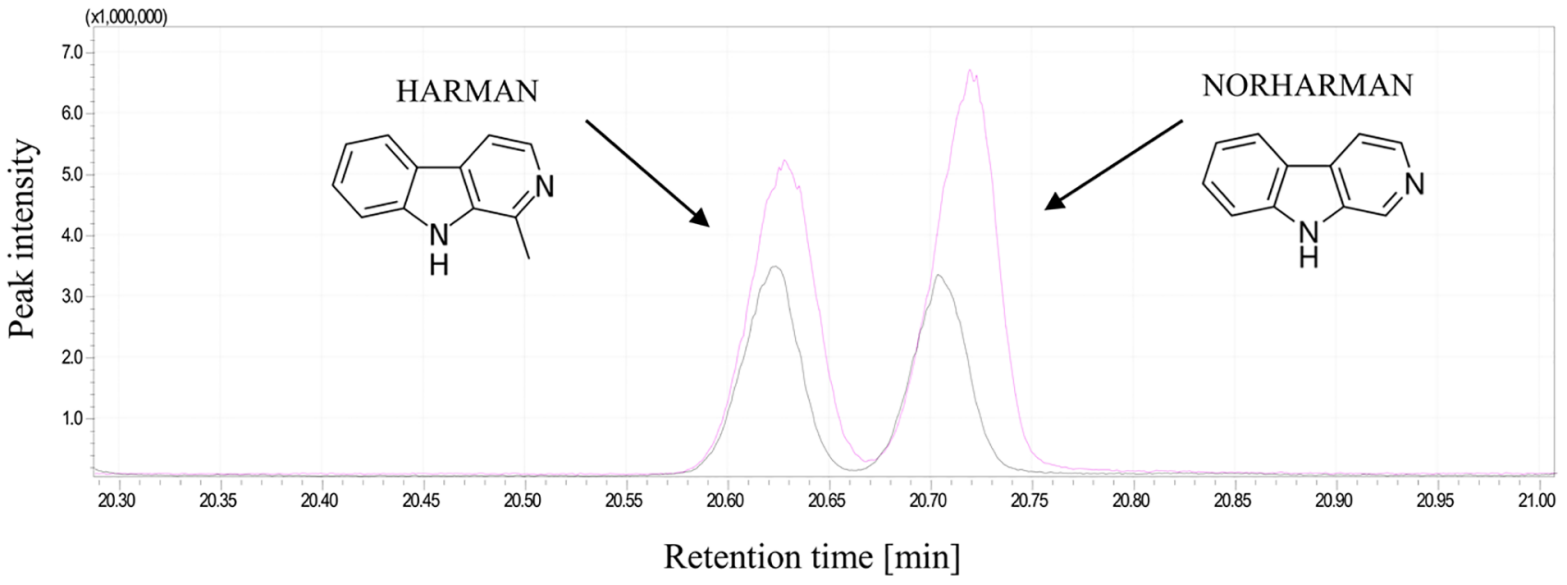
The total ion current (TIC) of harman and norharman extracted from MM (pink) and LB (black) post-incubation media.

The quantitative profiles of the harman and norharman produced by *C*. *coronatus* were found to depend on the choice of culture medium (Table 1; S1 Table). Fig 2 presents the total ion current (TIC) of harman and norharman extracted from both post-incubation media.

**Table 1:** Concentration of β-carboline alkaloids (μg/l ± SD) determined by GC-MS in cell-free filtrates of *C*. *coronatus*.

	MM medium	LB medium
**Harman**	6.91 ± 1.43 ^A^	3.49 ± 1.19 ^A^
**Norharman**	7.76 ± 1.67 ^B^	3.33 ± 0.82 ^B^

Statistically significant differences are marked with the same letters (Student’s t-test, P≤0.05).

*C*. *coronatus* cultured in minimal medium (MM) produced significantly more of both alkaloids compared with the fungus propagated in rich (LB) medium.

### Effects of harman and norharman on *G*. *mellonella* development

**Fig 3:**
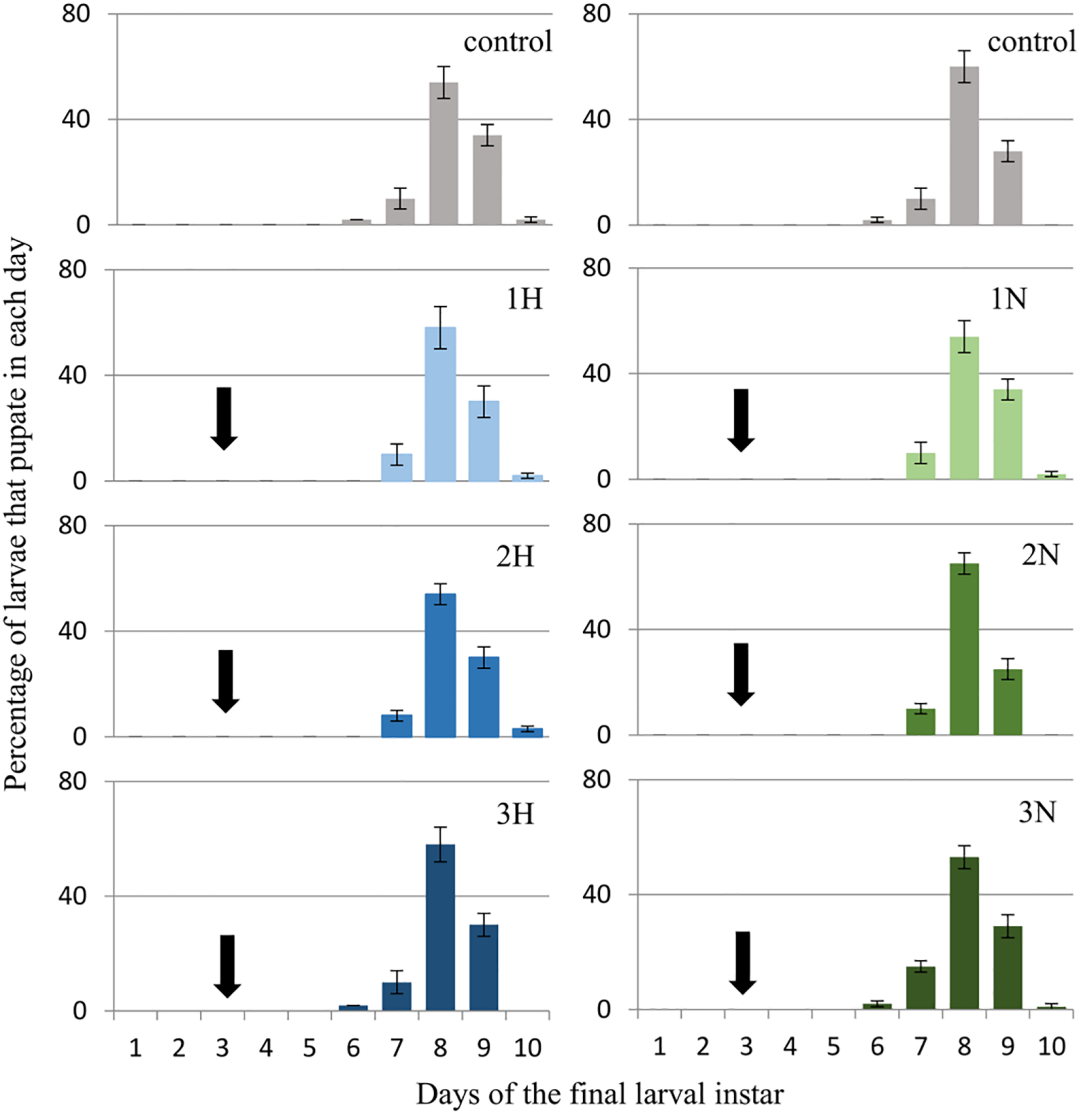
Effect of topical application of harman and norharman on pupation of *Galleria mellonella* larvae. 1H- harman 750 ppm, 2H- harman 1000 ppm, 3H- harman 1250 ppm. 1N- norharman 750 ppm, 2N- norharman 1000 ppm, 3N- norharman 1250 ppm. The day of administration of the alkaloids (final larval instar day 3) is marked with the arrow. In the 2H sample, 5% of the larvae died. In the remaining groups, all insects passed into the pupal stage.

**Fig 4:**
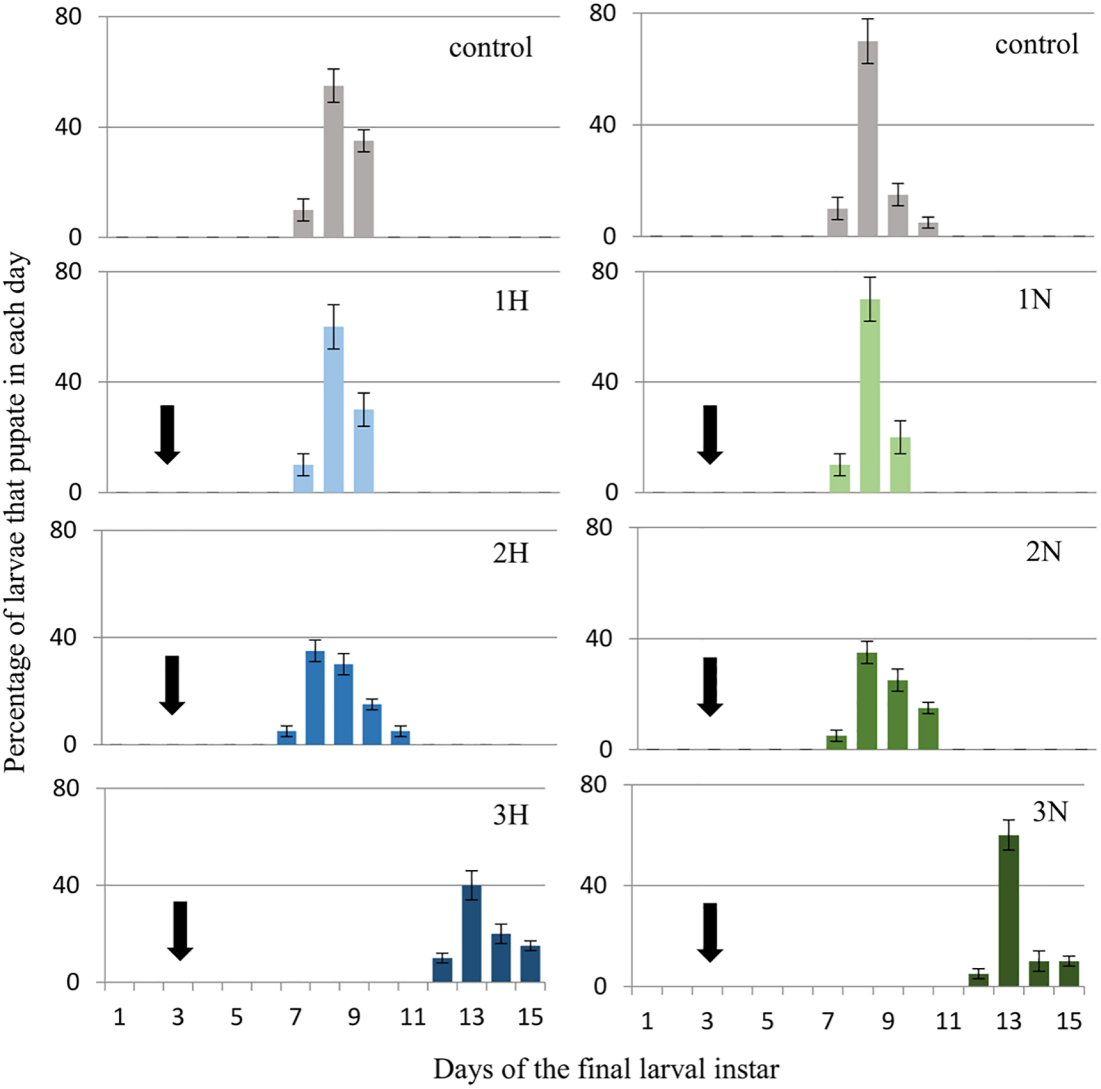
Effect of ingestion diet treated with harman and norharman on pupation of *Galleria mellonella* larvae. 1H- harman 750 ppm, 2H- harman 1000 ppm, 3H- harman 1250 ppm. 1N- norharman 750 ppm, 2N- norharman 1000 ppm, 3N- norharman 1250 ppm. The day of administration of the alkaloids (final larval instar day 3) is marked with the arrow. In the control, 1H and 1N groups, all larvae passed into the pupal stage. In the 2H, 3H, 2N and 3N groups, respectively 80%, 85%, 90% and 85% of larvae passed into the pupal stage.

**Fig 5:**
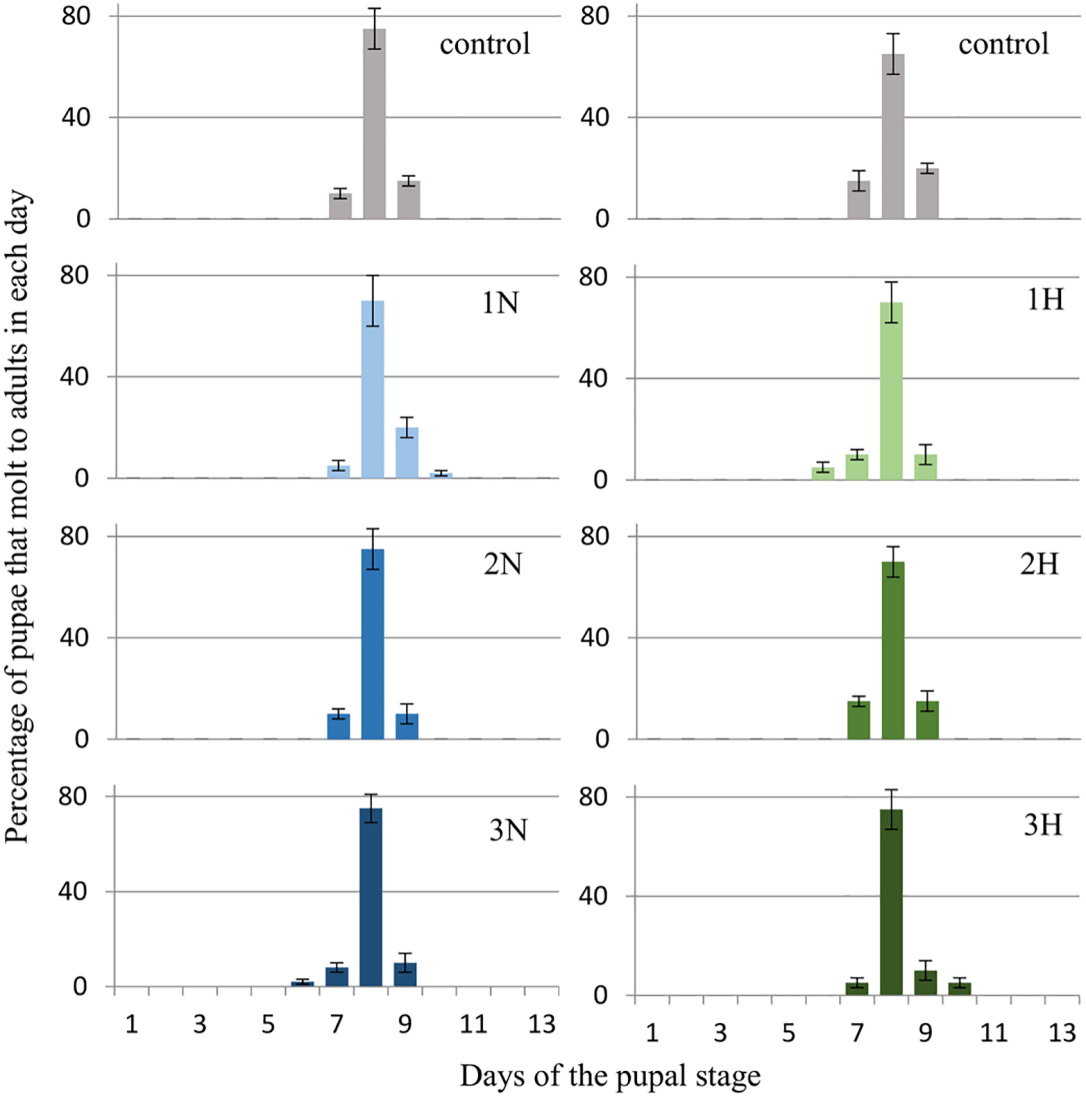
Effect of topical harman and norharman application on the adult molt of *Galleria mellonella* pupae. 1H- harman 750 ppm, 2H- harman 1000 ppm, 3H- harman 1250 ppm. 1N- norharman 750 ppm, 2N- norharman 1000 ppm, 3N- norharman 1250 ppm. Insects received alkaloids on the third day of the final larval instar. In controls and 2N groups, all pupae became adults. In the 1H, 2H, 3H, 1N, and 3N groups, respectively 97%, 95%, 95%, 95% and 95% of pupae became adults.

**Fig 6:**
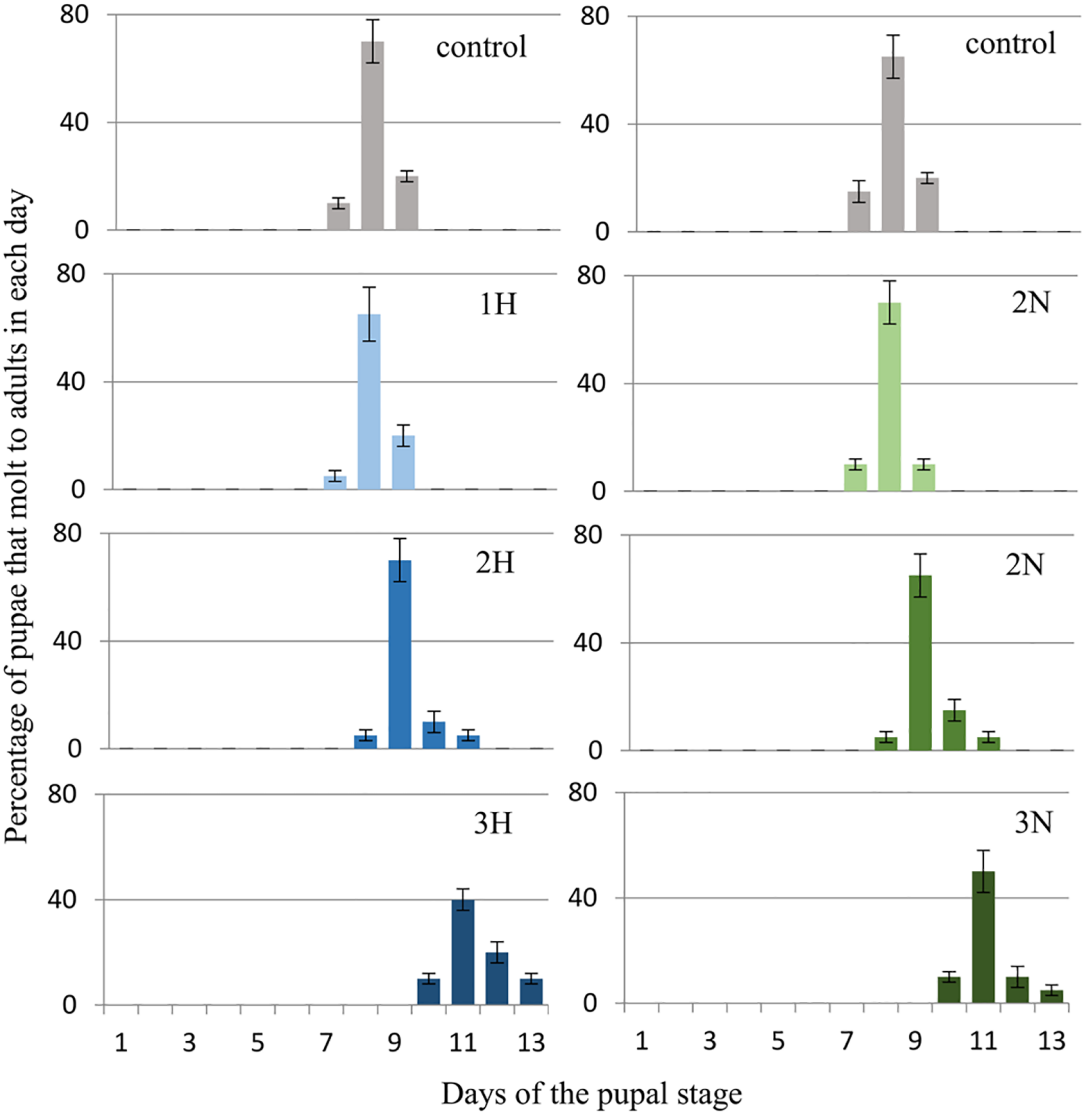
Effect of ingestion of harman and norharman with the diet on adult molt of Galleria mellonella pupae. 1H- harman 750 ppm, 2H- harman 1000 ppm, 3H- harman 1250 ppm. 1N- norharman 750 ppm, 2N- norharman 1000 ppm, 3N- norharman 1250 ppm. Insects received alkaloids on the third day of the final larval instar. In control groups, all pupae became adults. In the 1H, 2H, 3H, 1N, 2N and 3N groups, respectively, 90%, 90%, 80%, 90%, 90% and 75% of pupae became adults.

*G*. *mellonella* larvae (three-day-old last (7^th^) instar) were given harman (H) and norharman (N) topically and mixed with food at a final concentration of 750 (1H, 1N), 1000 (2H, 2N) or 1250 (3H, 3N) ppm. The effects of these compounds on pupation and adult eclosion are shown below. Figs 3 and 4 (see also S2 Table) show the percentage of larvae that become pupae on certain days, while Figs 5 and 6 (see also S3 Table) present the percentages of adults that emerged on certain days.

Topical administration of harman and norharman did not affect the pupation of *G*. *mellonella* larvae (Fig 3). No significant differences in mean pupation time were observed between the control group and insects treated with alkaloids.

Larvae (three-day-old last instar) were given alkaloids mixed with food. This method of administration had an impact on wax moth pupation (Fig 4).

Control larvae pupated on the eighth day of the final instar (8.03 ± 0.58 days); however, the larvae which ingested food mixed with alkaloids pupated one to four days later, depending on used concentration (1H: 8.1±0.6, 2H: 8.6±0.5, 3H: 12.7±1.1, 1N: 8.2±0.5, 2N: 8.5±0.6, 3N: 13.4±0.9 days). Statistically significant differences were observed between the control group and 3H group (P = 0.005) and 3N (P = 0.003).

Topical application of harman and norharman to three-day-old final instar larvae was not found to influence the emergence of *G*. *mellonella* adult (Fig 5).

Adult emergence was delayed when alkaloids were mixed with the diet (Fig 6). For the control group, emergence occurred 8.2±0.5 days after pupation. No statistically significant differences were observed between the control group and 1H (8.2±0.5 day), 2H (8.4±0.4 day), 1N (8.1±0.4 day) and 2N groups (8.2±0.6 day); however, significant differences were observed between controls and the 3H (11.5±0.9 day; P = 0.009) and 3N groups (12.2±1.1 day; P = 0.007).

### Effect of harman and norharman on serotonin and MAO-A concentration in *G*. *mellonella* heads

**Fig 7:**
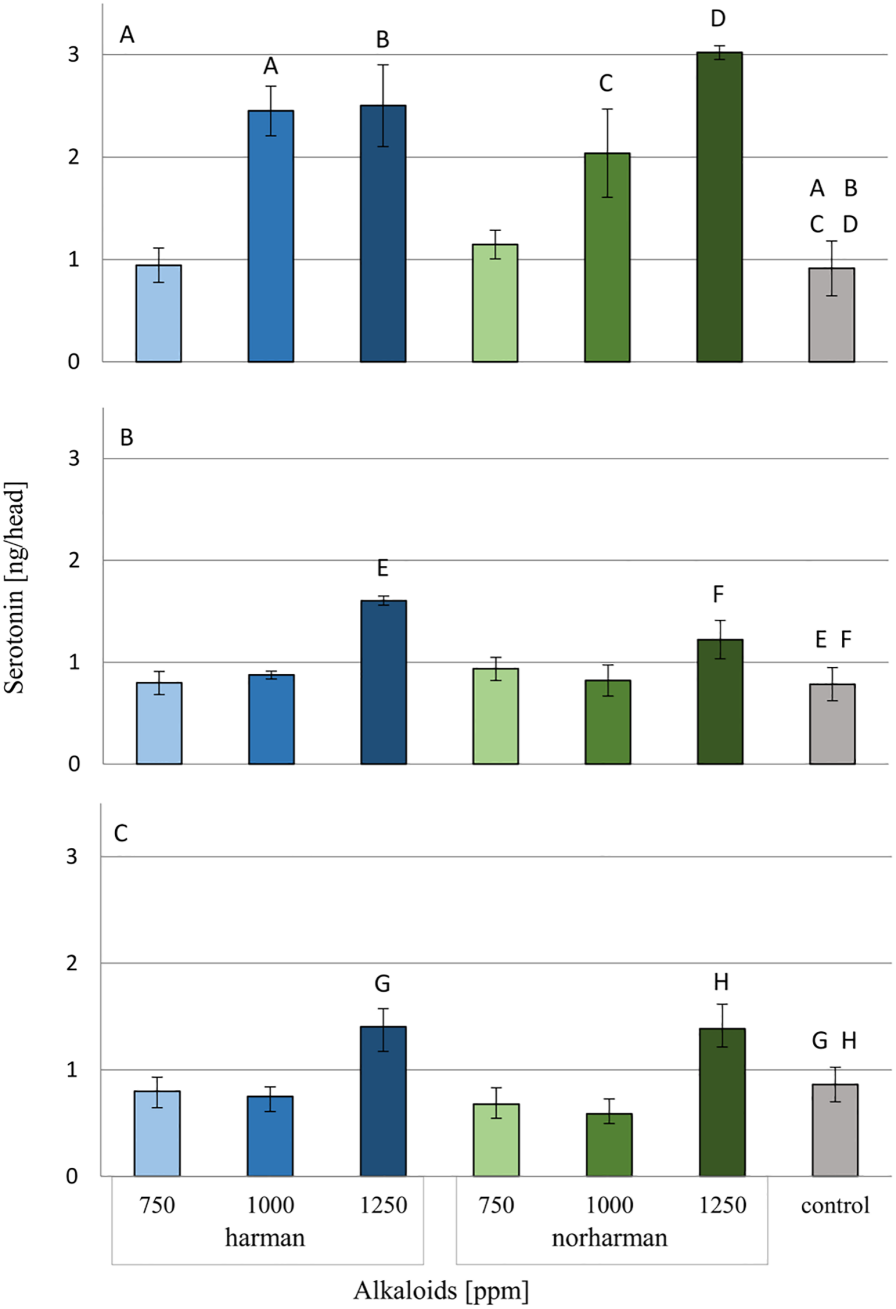
Serotonin concentration in heads of *G*. *mellonella* larvae treated with harman and norharman. A- One hour after topical application of alkaloids. B- 24 hours after topical application of alkaloids. C- 24 hours after ingestion of food mixed with alkaloids. statistically significant differences as compared to control was marked with the same letters, P≤0.05.

Serotonin levels in the heads were measured after topical application of harman and norharman to *G*. *mellonella* larvae or by ingestion. Fig 7 (see also S4 Table) shows the concentrations of serotonin in relation to the used doses of alkaloids.

Harman and norharman increased the serotonin concentration in the heads of the *G*. *mellonella* larvae. The greatest increase was observed one hour after topical application of both agents. In this group, a statistically significant (P<0.01) change was observed after exposure to concentrations of 1000 ppm and 1250 ppm. A statistically significant (P<0.001) increase was noted after 24 hours in groups that received 1250 ppm harman or norharman (topically and in food).

**Fig 8:**
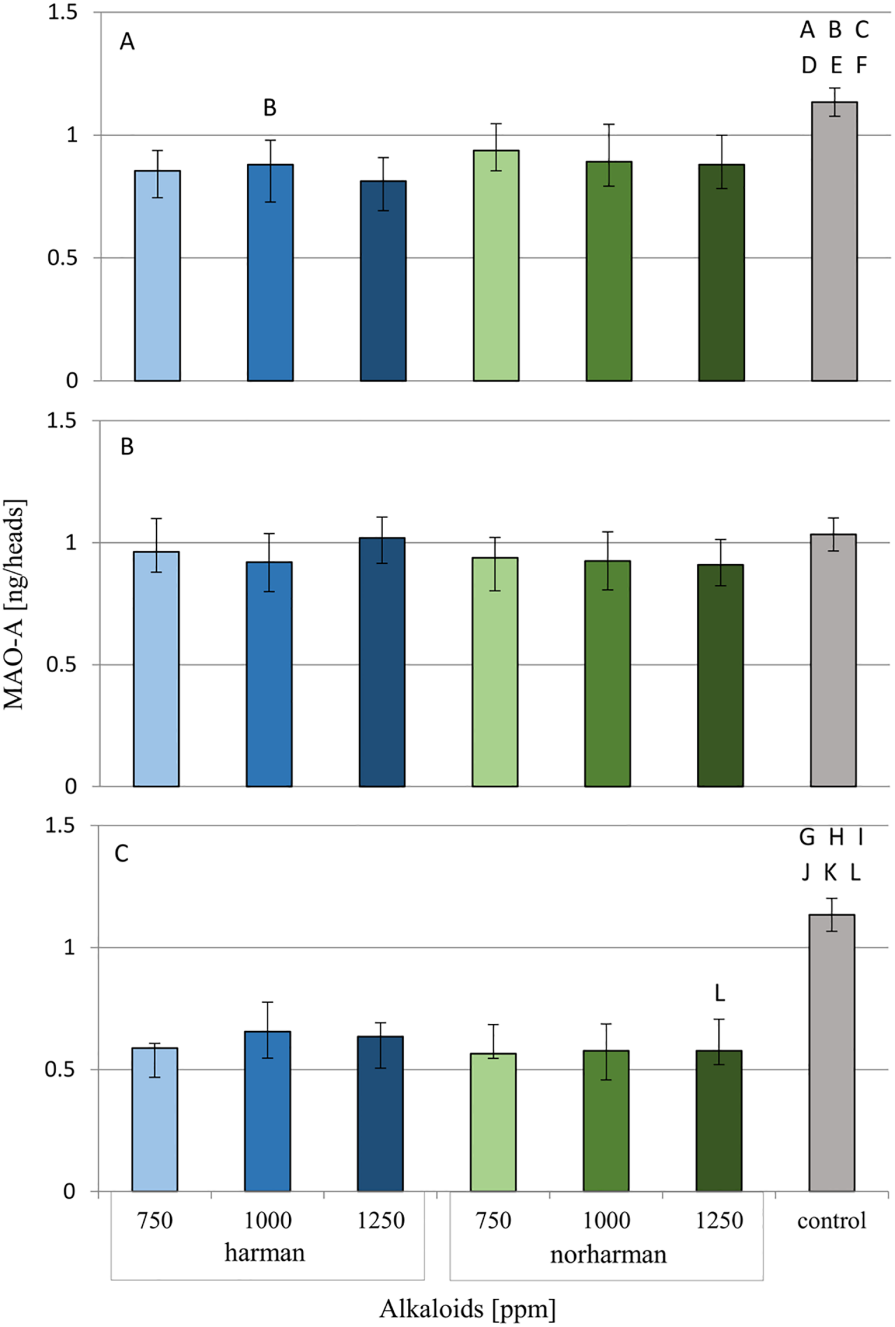
Effect of harman and norharman on MAO-A concentration in *G*. *mellonella* larvae heads. Data are expressed as mean ± SD. A- One hour after topical application of alkaloids. B- 24 hours after topical application of alkaloids. C- 24 hours after ingestion of food mixed with alkaloids. statistically significant differences as compared to control was marked with the same letters, P≤0.05.

The influence of harman and norharman on MAO-A levels in *G*. *mellonella* larval heads was examined. Its concentration in the examined tissues one hour and 24 hours after application of these compounds is given in Fig 8 (S5 Table).

No changes in MAO-A level was observed in the heads of *G*. *mellonella* larvae 24 hours after the topical application of harman and norharman. However, a statistically significant (P<0.01) decrease in MAO-A concentration in tissues was observed one hour after topical administration of alkaloids. The same effect was observed in the larvae that received test compounds with food for 24 hours.

### Effect of harman and norharman on MAO activity

**Fig 9:**
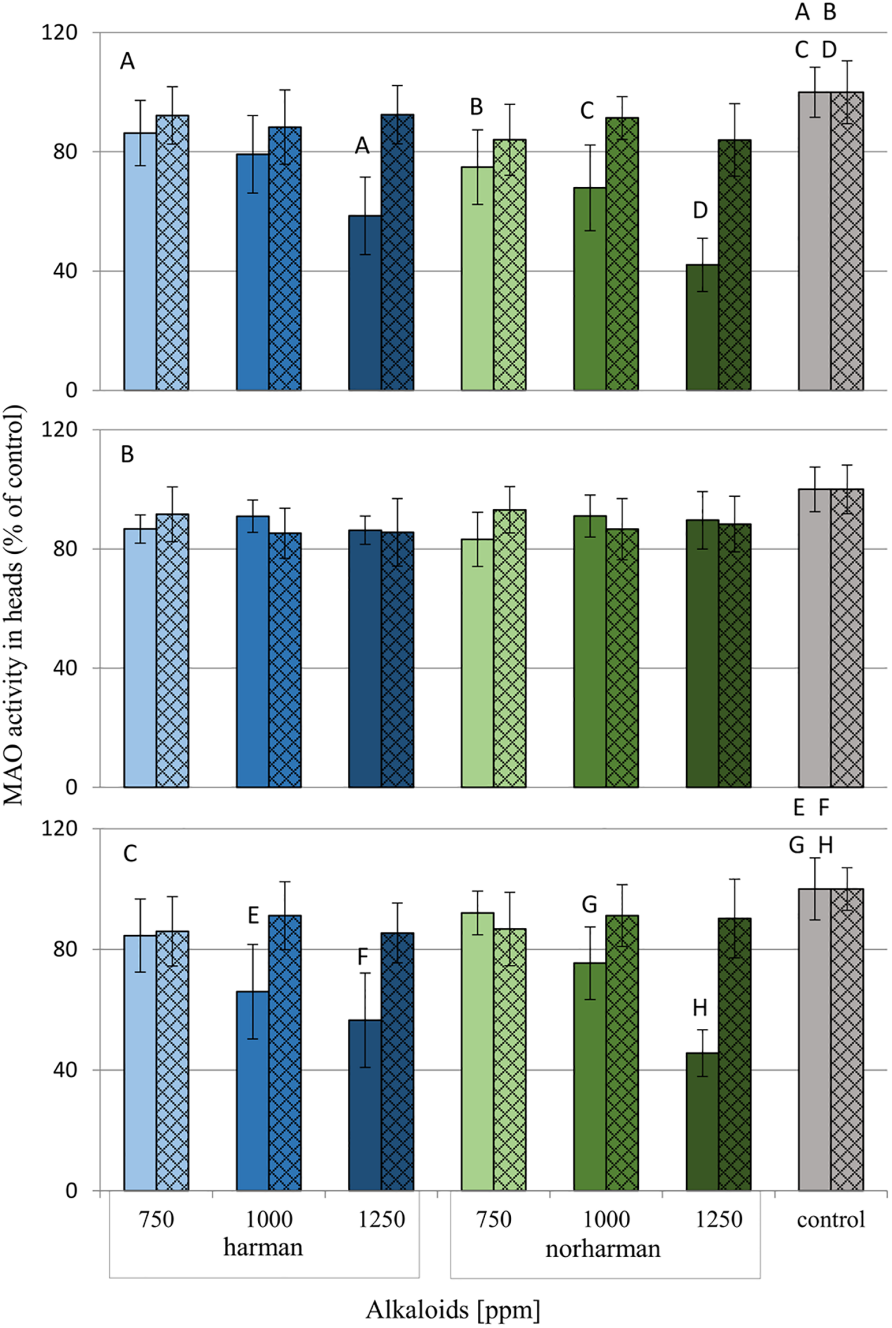
Effect of harman and norharman on MAO-A and MAO-B activity in the heads of *G*. *mellonella* larvae. Data are expressed as mean ± SD; empty bars- MAO-A activity; hatched bars- MAO-B activity. Data are presented as a percentage of the activity, control was taken as 100%. A-larvae after one hour from topical application. B- larvae after 24 hours from topical application. C- larvae after 24 hours from application with food. statistically significant differences as compared to control was marked with the same letters, P≤0.05.

The MAO-A and MAO-B activities in *G*. *mellonella* heads after harman and norharman application were examined. Values were given as percentages of activity relative to the control group, which was taken as 100%. (Fig 9; S6 Table).

Neither harman nor norharman was found to have any effect on MAO activity 24 hours after topical application. However, all concentrations (750, 1000 and 1250 ppm) of norharman and the highest concentration of harman (1250 ppm) were found to induce a decrease in the activity of both MAO-A and MAO-B after one hour. In addition, 24-hour administration of these alkaloids with food resulted in decreased MAO-A and MAO-B activity in the 2H, 3H, 2N and 3N groups.

## Discussion

The significant agents for regulation of insect populations are naturally-occurring entomopathogens. There are a few species of this organisms used for the control of insect pests [25].

The process of infection by an entomopathogenic fungus begins by adhesion to the body of the insect, and this is followed by the secretion of enzymes that hydrolyze the epidermis [26]. Death of the host occurs as a result of tissue destruction, exhaustion of nutrients or by the effect of fungal toxins. A number of toxic compounds such as small secondary metabolites, cyclic peptides and macromolecular proteins have been isolated from the filtrates of entomopathogenic fungi [27].

The toxic metabolites produced by entomopathogenic fungi have been reviewed extensively by Strasser et al. [28]. Among the fungal metabolites that assist pathogenicity are the destruxins of *Metharisium spp*. first described by Kodaira [29]. These toxins have been also detected in *Aschersonia sp*. [30]. Some strains of *Bauveria spp*. produce bauvericin, a dipeptide metabolite, which has demonstrated toxicity against a number of invertebrates [31], and which has also been isolated from *Paecilomyces fumosoroseus* and *Fusarium spp*. [27]. Two other toxins are linear peptidic efrapeptins, produced by *Tolypocladium* [32, 33].

*Conidiobolus coronatus* (Entomophthorales) is a saprophytic soil fungus that kills insects by releasing toxins inside the body of the insect, before invading the host organs and tissues with its hyphae [34]. It is pathogenic to a number of insects and can be relatively easily propagated under laboratory conditions [35]. The fungus is an interesting object to study and might also be a source of new insecticidal substances: Boguś and Scheller [4] found that 12 insect species, including all developmental stages of *G*. *mellonella*, were highly susceptible to *C*. *coronatus* infection.

However, not all the toxic fungal metabolites expressed by *C*. *coronatus* have been identified. Its culture filtrates and mycelial homogenates have been found to contain toxic proteins [4]; for example, a 30 kDa protein appeared to have very potent effect on *G*. *mellonella* larvae, with an LD 50 less than 5 ng/larva.

*C*. *coronatus* isolate 3491 was found to induce 100% mortality of *G*. *mellonella* last instar larvae exposed to sporulating colonies [7]. In addition, two other insecticidal proteins were identified in the post-incubation filtrates of the fungus: coronatin-1 and coronatin-2 [7, 8]. Coronatin-1, an insecticidal 36 kDa protein showing both elastolytic and chitinolytic activities, was then isolated by a two-step HPLC process [8].

A second protein, named coronatin-2 and weighing 4.5 kDa, was later isolated from *C*. *coronatus* and found to have toxic effects. However, in contrast to coronatin-1, it showed no enzymatic activity. When coronatin-2 was added to the hemocytes taken from the *G*. *mellonella* larvae, the nets formed by the granulocytes and plasmatocytes were observed to disintegrate. This could be attributed to the rapid degranulation of granulocytes and extensive vacuolization of plasmatocytes, as well as the expulsion of cytoplasm, and disintegration of the cells themselves. In addition, the spherulocytes were unaffected, but the oenocytes disintegrated rapidly. The injection of 5 μg/larva Coronatin-2 into *G*. *mellonella* resulted in 80% mortality [7].

A GC-MS analysis of fatty acids and amino acids isolated from *C*. *coronatus* by Gołębiowki et al. [35] also identified α- and β-glucopyranose. The identified fatty acids included 12–20, 22 and 24 carbon atoms per chain.

The GC-MS analysis conducted as part of the present study showed that *C*. *coronatus* produces two β-carboline alkaloids: harman and norharman. This is the first evidence this fungus has the ability to produce these substances. The highest amounts of norharman and harman were found in cell-free filtrates of MM post-incubation medium. As this medium is poor and provides only the basic ingredients for mycelium growth, it can be supposed that this paucity stimulates the fungus to produce larger quantities of toxic substances. For *Metarhizium anisopliae*, Shah et al [36] postulate that starvation conditions, whether *in vivo* or *in vitro*, result in de-repression of the protease Pr1, and that elevated levels of this enzyme enhance fungal virulence. The mechanisms underlying higher production of harmane and norharman by *C*. *coronatus* in starvation conditions (MM medium) remain unknown and need further experimental work. At present, it is unknown whether both alkaloids released by the growing fungus are directly involved in the pathogenesis and destruction of the insect host body induced by fungal infection. In the present study, the insects received harman and norharman in three concentrations according to Bouayad et al. [23]. However, the concentration of these alkaloids in the hemolymph and individual tissues of insects is currently unknown and is intended as the subject of further research.

Other fungi also produce alkaloids. *Aspergillus*, *Rhizopus*, *Penicillium* and *Calviceps* produce ergoline and erogotamine alkaloids. *Neosartorya tsunodane* produces indole alkaloids [37]. In addition, a marine sample of *Penicillium janczewski* was found to contain quinoline alkaloids [38].

β-carboline alkaloids, which are produced by *C*. *coronatus*, were originally isolated from *Peganum harmala*. Over the last two decades, numerous simple and complex β-carboline alkaloids, including harman and norharman, have been identified as major bioactive constituents from various terrestrial plants [39].

The effects of these alkaloids on mammals are well described. β-carboline alkaloids seem to interact selectively with specific enzymatic systems. Harman selectively inhibits apurinic/apyrimidinic endonuclease activity [40], and harman and other β-carbolines have been found to be potent inhibitors of 5-hydroxytryptamine-inducted human platelet aggregation [41]. In addition, norharman has been found to inhibit 2- acetylaminofluorence (AFF) N-hydroxylase, aldehyde oxidase, benzo(a)pyrene and harman cytochrome P450 [9].

These alkaloids have their most significant effects on the nervous system. Norharman and harman are known to be reversible competitive monamineoxidase (MAO) inhibitors: Norharman preferentially inhibits MAO-B, whereas harman inhibits MAO-A, and the two are considered to be the strongest inhibitors of MAO [42]. In addition to their interaction with enzyme systems, various receptor systems are also important protein targets for β-carboline. Since the first reports that β-carboline alkaloids are able to bind to serotonin (5-HT) receptors of isolated tissue [43], many investigations have examined this interaction. One study found that both harman and norharman bind to 5-HT receptors, causing an increase of 5-hydroxyindoleacetic acid (5-HIAA) and homovanillic acid (HVA) levels in rat brain [44].

Because serotonin and MAO are present in the insect nervous system, it can be hypothesized that harman and norharman also have an effect on this area. Our results show that these alkaloids increase the serotonin concentration in the heads of *G*. *mellonella* larvae. In addition, a statistically significant decrease in the concentration of MAO-A and total MAO activity was observed in tissues one hour after alkaloid administration. Therefore, it can be concluded that harman and norharman also act as MAO inhibitors in insects. Our results also show that these alkaloids have a negative effect on the pupation and adult emergence of *G*. *mellonella*, which may be due to elevated levels of serotonin.

Other studies have shown that the β- carboline alkaloids harman, harmine and harmaline have an effect on insects; however, this data has never been correlated with biomarkers present in the nervous system. Chronic dietary exposure tests (neonate to pupa) found harman to have potent antifeedant and possible toxic effects in *Spodoptera exigua* [45]. In addition, the presence of harmine in the larval diet evoked cannibalistic behavior and inhibition of pupation amongst *Plodia interpunctella* larvae [23]. Finally, harman and harmaline reduced growth and feeding in the fifth-instar larvae of *Trichoplusia ni* [46].

Our findings indicate that 24 hours of exposure to food contaminated with harman and norharman and one hour of topical application influenced the activity of *G*. *mellonella* larvae. However, the finding that these alkaloids lost their effects following 24 hours from topical application may suggest that they are metabolized and/or excreted by insects. It has been suggested that in humans, β-carboline alkaloids are metabolized in the liver [47]; during this process, β-carbolines are efficiently oxidized to several ring-hydroxylated and N-oxidation products with cytochrome P450 playing a role [48]. The mechanisms of harman and norharman metabolism in insects are unknown.

The results of this innovative study may have a considerable impact on research into insect physiology, parasitology, mycology and biocontrol of pests. These studies confirm for the first time that the parasitic fungus *C*. *coronatus* produces two β-carboline alkaloids (harman and norhaman) *in vitro*, and reveal they disorder insect development and influence serotonin-regulating enzymes. Our findings provide further knowledge about the potential role of these alkaloids in the process of fungal infection, which may lead to the development of more effective and environmentally-friendly means of controlling insect pests.

## Supporting information

S1 Table Concentration of β-carboline alkaloids (μg/l) determined by GC-MS in cell-free filtrates of *C*. *coronatus-* raw data. (XLSX)

S2 Table Effect of harman and norharman topical application and ingestion with food on pupation of *Galleria mellonella* larvae- raw data. (XLSX)

S3 Table Effect of harman and norharman topical application and ingestion with food on adult molt of *Galleria mellonella* pupae- raw data. (XLSX)

S4 Table Serotonin concentration in heads of *G*. *mellonella* larvae treated with harman and norharman- raw data. (XLSX)

S5 Table Effect of harman and norharman on MAO-A concentration in *G*. *mellonella* larvae heads- raw data. (XLSX)

S6 Table Effect of harman and norharman on MAO-A and MAO-B activity in G. mellonella larvae heads- raw data. (XLSX)
